# The Roles of T cells in Immune Checkpoint Inhibitor-Induced Arthritis

**DOI:** 10.14336/AD.2024.0546

**Published:** 2025-07-26

**Authors:** Maike Chen, Huili Li, Baicheng Qu, Xin Huang

**Affiliations:** ^1^Department of Orthopaedics, Union Hospital, Tongji Medical College, Huazhong University of Science and Technology, Wuhan 430022, China.; ^2^Department of Thoracic Surgery, Union Hospital, Tongji Medical College, Huazhong University of Science and Technology, Wuhan 430022, China.

**Keywords:** Immune checkpoint inhibitor, Arthritis, Immune-related adverse events, T cells

## Abstract

Immune checkpoint inhibitor (ICI) therapy, a novel anti-tumor strategy, can specifically eliminate tumors by activating the immune system and inhibiting tumor immune escape. However, ICI therapy can lead to notable negative outcomes known as immune-related adverse events (irAEs). ICI-induced arthritis, also known as ICI arthritis, stands as the prevailing form of irAEs. The purpose of this review is to highlight the crucial functions of T cells in the progression of ICI arthritis. Under the influence of different signaling molecules, T cells could gather in large numbers within the synovial membrane of joints, releasing inflammatory substances and enzymes that harm healthy tissues, ultimately causing ICI arthritis. Moreover, considering the functions of T cells in triggering ICI arthritis, this review suggests several treatments to prevent ICI arthritis, including inhibiting the overstimulation of T cells at the synovial sac of joints, enhancing the precision of ICI medications, and directing ICI drugs specifically towards tumor tissues instead of joints. Collectively, T lymphocytes play a vital role in the onset of ICI arthritis, offering a hopeful perspective on treating ICI arthritis through the specific targeting of T cells within the affected joints.

## 1. Introduction

**Figure 1.**
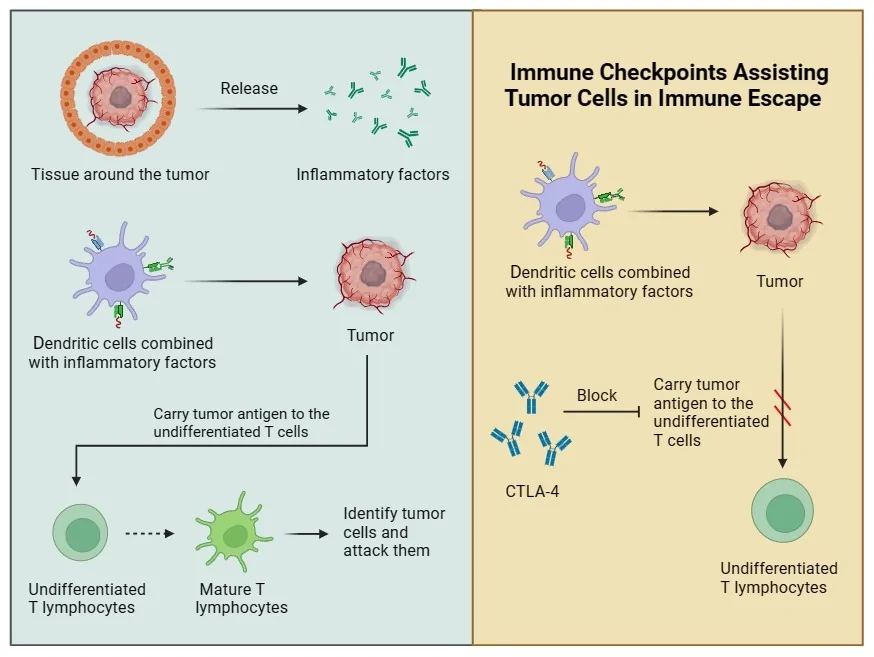
**Mechanisms of immune checkpoints assisting tumor cells in immune escape**. CTLA-4 is a typical type of immune checkpoint, and its function lies on preventing DC cells from carrying tumor antigens and activating juvenile T lymphocytes. Once CTLA-4 is turned on, DC cells could not present tumor antigens to T cells, and T cells could not recognize and further kill tumor cells.

Immune checkpoints refer to small molecules present on the surface of immune cells that impede the immune cells’ ability to recognize and eliminate threats. Common immune checkpoints include CTLA-4 (Cytotoxic T-Lymphocyte-Associated protein 4), PD-1 (Programmed Death 1), KIR (Killer-cell Immunoglobulin-like Receptor), and others [1]. They inhibit the proliferation of immune cells and even induce their apoptosis. Tumor cells can utilize these immune checkpoints to achieve immune escape [1]. For example, CTLA-4, located on the surface of juvenile T lymphocytes, functions to prevent dendritic cells from carrying tumor antigens and stimulating the activation of juvenile T lymphocytes [2, 3]. After the activation of CTLA-4, the transmission of tumor antigens from DC cells to T cells is hindered, resulting in the inability of T cells to identify and eliminate tumor cells (Fig. 1).

Immune checkpoint inhibitors (ICIs) have the ability to inhibit immune checkpoints, leading to the elimination of tumor cells by the immune system. The current ICI therapy might inhibit immune checkpoints on the cell surface by directly binding to them and keep immune cells in an activated state [4, 5]. Motivated immune cells could further recognize and eliminate tumor cells [6]. For instance, CTLA-4 inhibitor can specifically prevent CTLA-4 from functioning, thereby enabling profitably activation of undifferentiated T cells [7] (Fig. 1). The outlook for patients with tumors has greatly improved due to the rapid advancement of ICIs.

The effectiveness of ICI treatment has been widely recognized in the world. Nevertheless, it has been widely reported that ICIs can cause excessive activation of the immune system, resulting in various immunological issues referred to as immune-related adverse events (irAEs) [8-11]. Some of the most frequent irAEs are myocarditis, neurological complications, and ICI-induced arthritis [9, 12, 13]. Current research indicates that ICI arthritis is mainly triggered by the activated T cells in synovial tissues. This special type of arthritis could harm the joint continuously. The joint pain and mobility inconvenience triggered by it bother the patients seriously [14]. Due to its unique nature, the common arthritis therapies are not applicable for patients with ICI arthritis [12]. The occurrence of ICI arthritis limits the widespread use of this excellent ICI treatment for tumors.

It is therefore necessary to conduct further research on ICI arthritis, which shares numerous similarities with autoimmune arthritis. In terms of etiology, both conditions are caused by an overly excited immune system attacking joint tissue. Research has shown that, like autoimmune arthritis [15], T cells play a crucial role in the progression of ICI arthritis [16], being the primary cells responsible for carrying out the immune system’s attack mechanisms.

Hence, the objective of this review is to elucidate the functions of T cells in ICI arthritis through the exploration of the etiology of ICI arthritis and its juxtaposition with primary arthritis. The unique role of T cells is crucial to propose possible treatment strategies for ICI arthritis. Furthermore, more in-depth investigations are warranted for the treatment of ICI arthritis.

## 2. The possible pathogenesis of ICI arthritis

The pro-inflammatory impacts of T cells in osteoarthritis at the molecular level are mainly due to various cell subtypes, such as Th1, Th9, and Th17 [17]. These subtypes of cells are the primary source of pro-inflammatory cytokines, while TNF-α, IL-17 and other cytokines can induce chondrocyte apoptosis, inhibit cartilage synthesis and promote cartilage degradation, which results in destructive effects on joints. [17-19]. Furthermore, research findings suggest that, following ICI treatment, activated macrophages and T cells proliferate extensively in the blood, subsequently infiltrating the joints from local blood vessels and producing a range of cytokines, notably tumor necrosis factor (TNF) [19]. The proteases secreted by activated macrophages have been observed to degrade ligaments, tendons, and bones. Concurrently, activated T cells may secrete a multitude of pro-apoptotic factors, which could potentially induce apoptosis in normal tissues within the joint. Additionally, T cells are capable of producing lactic acid through aerobic respiration, which can result in toxic effects on tissue cells [19, 20]. Accordingly, from the molecular standpoint, the excessive secretion of cytotoxic cytokines, proteolytic enzymes, and lactate by the immune system represents a key etiological factor in the pathogenesis of ICI arthritis.

It has been suggested that the development of ICI arthritis at the cellular level could be linked to the stimulation of CD8+ T lymphocytes and the resulting decrease in regulatory T cells (Treg cells). Observations revealed that patients with ICI arthritis exhibited a higher number of T cells and a lower number of Treg cells, which can be attributed to the immune activation effects of Cistern cells, also known as regulatory T cells. This specific group of cells has a crucial function in controlling the body’s immune response [20]. CD8+ T lymphocytes exhibit strong aggressiveness and are essential for carrying out cellular immune responses in the body [21]. An overabundance of CD8+ T cells have the potential to attack and destroy normal tissue cells, resulting in tissue damage and organ dysfunction [22]. Regulatory T cells, also known as Treg cells, have the ability to suppress the immune response of other cells by contacting them and releasing inhibitory cytokines like Foxp3, CD25, and CD4 in different subsets of immune cells, ultimately serving to regulate the immune response in a negative manner within the body. The depletion of regulatory T cells (Treg cells) may result in the alleviation of autoimmune diseases, whereas their expansion may lead to an exacerbation of tumor progression [20]. It can be posited that the accumulation and expansion of activated CD8+ T cells and the reduction of Treg cells in the synovial tissues may be responsible for the development of ICI arthritis.

The complete process of the onset of ICI arthritis is presented based on the aforementioned levels. Following ICI treatment, there is an extensive proliferation of activated macrophages and T cells in the blood, which subsequently penetrate the joints from local blood vessels. The proteases secreted by activated macrophages have the capacity to degrade ligaments, tendons, and bones. Concurrently, activated T cells may secrete a multitude of pro-apoptotic factors, which could potentially induce apoptosis of normal tissues within the joint. Additionally, T cells are capable of producing lactic acid through aerobic respiration, which can result in toxic effects on tissue cells.

Actually, the development of ICI arthritis is a complex process involving excessive protease production, apoptosis-promoting factors, and a large number of harmful substances produced by the activated immune system. Additionally, ICI arthritis is associated with the patient’s inherent biological predispositions and their lifestyle choices. Congenital conditions include the presence of genes encoding the five amino acid sequences of the HLA-DR4 hypervariable region, PTON22 polymorphisms, and so forth [23]. Consequently, some patients are more susceptible to developing ICI arthritis due to underlying physiological factors [24, 25]. Recent studies have also found possible additional elements, such as levels of estrogen, smoking habits, viruses, bacteria, and other microorganisms, that could impact the human immune system and play a role in the onset of ICI arthritis [26-28].

## 3. The clinical differences between ICI arthritis and autoimmune arthritis

From both a pathogenetic and cellular perspective, ICI arthritis has been observed to exhibit similarities with autoimmune arthritis [29]. Accordingly, an analysis of the distinctions between these two conditions may facilitate the identification of the distinctive characteristics of ICI arthritis and the development of potential treatment strategies.

### 3.1. Differences in the affected area

Autoimmune arthritis, like rheumatoid arthritis (RA), typically manifests in the small joints of the hands and feet, particularly the proximal interphalangeal joints, metacarpophalangeal joints, and metatarsophalangeal joints. Subsequently, it is frequently observed in the knee, ankle, and other joints [30, 31]. The sites of onset are invariably symmetrical. Autoimmune arthritis is primarily caused by the malfunction of synovial membranes, resulting in the deterioration of articular cartilage and joint capsule, which ultimately leads to joint ankylosis deformity. Furthermore, ICI arthritis is characterized by significant symmetry, with a propensity to affect the shoulder, knee, hip, and metacarpophalangeal joints [15, 32, 33]. The onset of ICI occurs between five and 6.5 months following the administration of ICI therapy. Additionally, it is primarily attributable to synovial membrane lesions. In conclusion, autoimmune arthritis and ICI arthritis exhibit comparable patterns of involvement [25].

### 3.2. Differences in gender ratio

The male to female ratio of patients with autoimmune arthritis is approximately 1:4, and this variation in the male-to-female ratio could be attributed to changes in hormone levels. After entering climatarian, the level of sex hormones in women decreases faster and more severely, which is related with autoimmune arthritis firmly. In contrast to autoimmune arthritis, there is no substantial gender disparity observed in ICI arthritis. While the incidence of women following ICI treatment for specific tumors is comparatively higher than that of men, this phenomenon is not statistically significant [25].

### 3.3. Differences in age composition

It is crucial to highlight the significant difference in age distribution between the two illnesses. From the perspective of pathogenesis, there are no significant external factors that induce autoimmune arthritis. Rather, its effects accumulate over time [32], so it is a common occurrence in middle-aged and elderly individuals. ICI arthritis is mainly attributed to strong external factors, leading to a broader spectrum of ages among patients with the condition. Furthermore, the disparate levels of immune system activity associated with age-related differences give rise to significant variations in symptom presentation. The inflammatory response is significantly more pronounced in younger individuals than in older individuals [32]. This could be attributed to the heightened immune activity observed in younger patients [34] (Table 1).

It can be seen that ICI patients span a wider age range, with no significant gender differences. Consequently, the treatment of ICI arthritis will be more complex, and it is necessary to consider developing corresponding treatment strategies for patients of different age groups.

**Table 1:** Clinical difference between ICI arthritis and autoimmune arthritis.

	Autoimmune arthritis	ICI arthritis
**Constitutional symptom**	Fever Asthenia Weight loss Malaise Anorexia Arthralgias Myalgias	Fever Arthralgias Myalgias Aphasia Facial swelling Dysphagia Myasthenia
**Pathogenic site**	Metacarpophalangeal joint Proximal interphalangeal joint Wrist joint	Metacarpophalangeal joint Proximal interphalangeal joint Wrist joint Phalangeal joint Enthesitis
**Sex related or not**	Male to female: about 1:4	No significant difference
**Age related or not**	No significant difference	A much stronger inflammatory response in youngsters
**Rheumatic autoantibodies**	Detected	Detected

## 4. The features of T cells in individuals with ICI arthritis

### 4.1. Imbalanced T cell composition ratio: more CD8+ T cells and less Treg cells

Patients with ICI arthritis exhibit a significant elevation in specific T cell subsets, alongside a decline in other T cell subsets, indicating a dysregulation in immune cell composition.

Patients with ICI arthritis had their CD8+ T cells sorted in a study, followed by conducting paired single-cell antigen receptors and transcriptome sequencing. Researchers identified 8 clusters with distinct transcriptions from a pool of 18472 synovial CD8+ T cells, revealing that the predominant cluster (GZM+ cluster) displayed notable cytotoxicity. The abundance of these GZM+ populations was attributed to the extensive clonal expansion of locally proliferating cells in ICI arthritis joints [18]. Patients with ICI arthritis had significantly higher levels of CD8+ T cells in their synovial fluid compared to those with RA or psoriatic arthritis. Additional studies have been carried out on CD8+ T lymphocytes in individuals with ICI-induced arthritis, leading to the identification of distinct characteristics of CD8+ T cells in ICI arthritis. PD-1 and Ki67 (a protein amount linked to cell division) were identified on CD8+ T cells. Various CD8+ T cell groups, including effector cells, memory cells, and cytotoxic CD8+ T cells, were observed in synovial tissue and fluid. Among them, cytotoxic CD8+ T cells have the highest level, and they exhibit functional impairment and high proliferative activity [35].

The researchers employed flow cytometry to quantify T cell subsets at various time points throughout the course of ICI therapy. The first objective was to ascertain the frequency of typical Tregs expressing CD4, CD25, and FOXP3. A decrease in Tregs was noted during irAE, while patients without adverse events did not show any changes in Tregs while receiving ICI treatment. Additionally, the Tregs frequency at the same time points was notably reduced in the irAE group compared to the non-irAE group [20]. Regulatory T cells have the ability to inhibit the immune response of different cells by contacting them and releasing suppressive cytokines like Foxp3, CD25, and CD4 in different subsets of immune cells. This negative regulatory role is crucial in maintaining immune homeostasis within the body [36].

**Figure 2.**
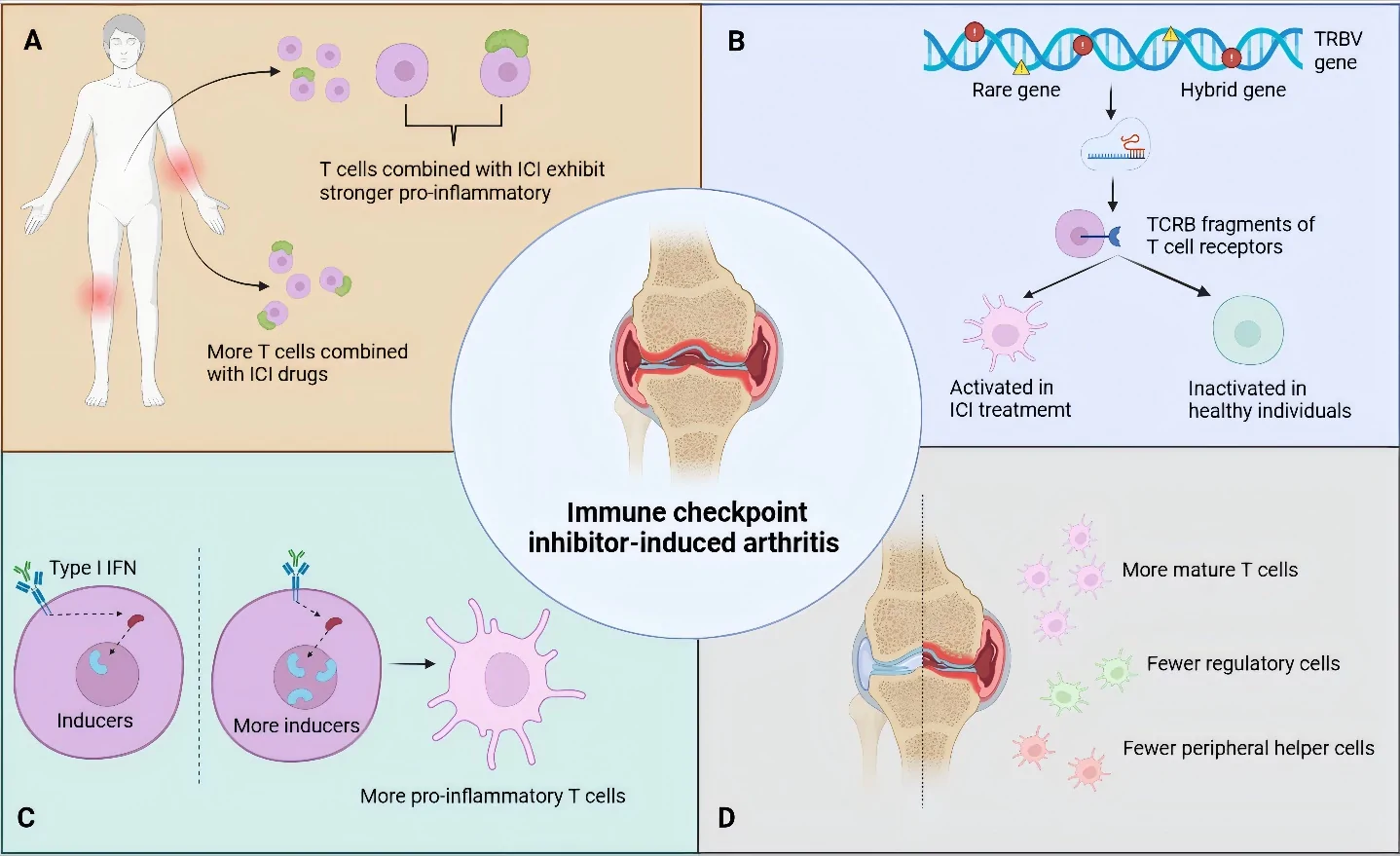
**The characteristics of T cells in patients with ICI arthritis**. (**A**) More T cells bind to ICI drugs at the synovial capsule of the joint with stronger activity; (**B**) Some upstream genes in the TCRB region of T controlled cell surface receptors are disrupted. These cells are dormant in the normal body and activated during ICI treatment; (**C**) T cells in ICI arthritis are more sensitive to type I IFN and more likely to be induced into toxic T cells; (**D**) Patients with ICI arthritis have more mature T cells and fewer T peripheral helper cells and regulatory cells in the synovial fluid.

To sum up, individuals with ICI arthritis exhibit a unique change in the cellular makeup of their immune system. A significant rise in CD8+ T cells exhibiting high cytotoxicity is observed, along with a marked decrease in Treg cells known for their immunosuppressive properties. This change in the cells provides insight into the immune system malfunction in individuals with ICI arthritis (Fig. 2D).

### 4.2 Imbalanced T cell gene expression: more CD38 and HLA-DR expression, less CD127 expression

Besides the dysregulation of T cell composition, the dysregulation of T cell gene expression is also a significant factor contributing to immune system disorders in patients with ICI arthritis. The unique gene expression pattern of T cells in individuals with ICI arthritis is a crucial distinguishing feature that separates it from autoimmune arthritis.

The T cell phenotypes of ICI arthritis patients were compared with those of RA and PsA patients using mass spectrometry. The findings indicated an increase in the levels of CD38 and HLA-DR expression in T cells of patients with ICI arthritis, along with a decrease in CD127 expression [35]. The dysregulation of gene expression in T cells can have many negative effects on T cells, further leading to immune dysfunction in the body. (a) CD38 can reduce the mitochondrial function of CD8+ T cells, decrease their activity and regeneration. Simultaneously, CD38 hinders the expansion, cytotoxicity, and cytokine release of CD8+ T cells via the adenosine pathway, resulting in reduced responsiveness to PD-1/PD-L1 antibody treatment [37]. (b) HLA-DR typically appears only in the advanced phase of T cell activation in response to the immune system. The causes and outcomes of the increase in HLA-DR expression in T cells have not been thoroughly examined, however, there is strong evidence indicating that this is a characteristic feature of T cell impairment [38]. (c) CD127 is crucial in the maturation of thymocytes to T lymphocytes, the balance of mature T cell levels, the immune reaction of T cells post viral infection, and the formation and longevity of memory T cells. The decrease in CD127 expression level indicates the disruption of the internal homeostasis of T cells and possible loss of cellular function [39].

To sum up, patients with ICI arthritis might exhibit abnormal gene expression specific to T cells, characterized by increased CD38 and HLA-DR expression and reduced CD127 expression. Gene expression abnormalities can disrupt the typical operation of T cells, leading to immune system dysfunction in individuals with ICI arthritis.

### 4.3. Some special T cell surface receptors lead to higher incidence rate of ICI arthritis

Studies show that some individuals might be more prone to developing irAEs after receiving ICI therapy because of specific T cell receptors on their surface. These receptors are linked to individual genetic variations, leading to the activation of dormant T cells in the body.

The complementarity-determining region (CDR) is a region on the T-cell receptor (TCR) whose structure determines its antigenic specificity. The T-cell receptor beta variable (TRBV) gene encodes the CDR [40]. Individuals with germline variants of TRBV may be susceptible to T cell dysfunction. The presence of these differences also impacts the interaction between TCR and human leukocyte antigen (HLA), thereby enhancing the likelihood of recognizing self-antigens [40].

Prior research investigated the connection between TRBV diversity and severe irAEs by studying blood samples from 81 patients who encountered varying degrees of irAEs while undergoing ICI therapy [40]. In each sample, researchers create allele profiles based on the TCRB receptor library, and then divide each allele profile into haplotypes, which are six kinds of allele profiles. The authors observed significant differences between the six haplotype groups when it came to the incidence of severe irAEs: among patients from one haplotype group, severe irAE was less common, whereas among patients from the other haplotype groups, severe irAE occurred in 14-44% of cases [40]. It indicates that there is indeed a connection between high-risk or low-risk group for ICI arthritis and their TCR [41]. In fact, anther experiments further showed that patients with TCR genome mutations are at high risk for ICI arthritis [40]. These T cells with specific TCR have common genetic characteristics: they have mutations in the TRBV region genes encoding TCRs, with more alleles and rare genes [41, 42]. On the contrary, T cell populations with higher homozygosity of TRBV region genes exhibit high resistance to irAE (Fig. 2B).

It is hypothesized that the mutation of T cell surface receptors is a contributing factor to the elevated incidence of irAEs. The proposed mechanism involves the disruption of TCR receptors on the surface of these T cells, leading to their inhibition and inactivation in normal organisms. Nevertheless, ICI therapy may revive these T lymphocytes, leading to a notable rise in their quantity after receiving ICI treatment. Concurrently, the confusion of these surface receptors on the cells increases the likelihood of autoimmune reactions. This explains the significant increase in T cells after ICI treatment, which can lead to autoimmune reactions such as ICI arthritis because of the higher T cell infiltration [22, 40].

### 4.4. T cells exhibit higher IFN sensitivity at the joints

Interferons (IFN) are proteins produced by the immune system that can penetrate T cells and influence their transcription by activating transcription factors within the cells, thereby facilitating the development and specialization of T cells [43]. It has been demonstrated that T cells in patients with ICI arthritis exhibit heightened sensitivity to the inducer type I IFN in comparison to T cells in RA [22].

In a study of over 1,000 transcription factors, it was discovered that in ICI arthritis T cells, only eight transcription factors were increased compared to RA or PsA Among these eight, three were common elements of transcription factor groups activated by type I IFN [22, 44]. In other words, in comparison to autoimmune arthritis T cells, ICI arthritis T cells demonstrate greater sensitivity to type I IFN.

Concurrently, research has demonstrated that stimulation of type I IFN can also induce monocyte differentiation in patients with RA into T cells with the characteristics of ICI arthritis [22, 43]. Synovial monocytes from patients with RA or PsA were treated with interferon-β (type I IFN) or interferon-γ (IFN-γ) in vitro by the scientists. Compared with interferon-γ, interferon-β induced RA and PsA synovial monocytes to differentiate and acquire T cell phenotypes of ICI arthritis. Furthermore, in comparison with T cells in ICI arthritis, these induced cells demonstrate elevated expression of perforin, granzyme B, and Ki67.The induced mature T cells exhibit heightened bioactivity, thereby enhancing their capacity to effectively eliminate target cells. Type I IFN has been demonstrated to markedly enhance the cytotoxic capacity of primitive, immature T cells (Fig. 2C). It is noteworthy that subsequent studies have demonstrated that, despite the increased prevalence of fully developed T cells in the peripheral blood of individuals with ICI arthritis, the elevation in IFN traits has not been consistently observed. This suggests that the activity of IFN may be confined to the affected joints.

In summary, patients with ICI arthritis show heightened sensitivity of T cells to type I IFN, suggesting an increased likelihood of developing mature, highly cytotoxic T cells upon exposure to type I IFN. Consequently, the targeted inhibition of IFN at the affected joints may potentially mitigate the severity of ICI arthritis in patients, as detailed in the following section.

### 4.5. ICI drugs bind to T cells and coexist in the bloodstream and synovial fluid of joints

Patients with ICI arthritis exhibit a unique characteristic in their T cells, which show specific affinity towards ICI medications and can be found in both the blood and synovial fluid through this interaction. This binding is highly specific and is determined by the phenotype and location of T cells [22].

Firstly, the binding of ICI drugs to T cells has been observed to exhibit phenotype specificity. Related studies have demonstrated that the majority of circulating ICI drugs bind to T cells that exhibit typical ICI arthritis phenotypes, namely CD38 high expression and CD127 low expression T cells. Hence, we hypothesize that the attachment of these ICI medications to T lymphocytes could potentially contribute to the disruption of T lymphocyte genetic activity. Secondly, the binding of ICI drugs to T cells has positional specificity. CD8+ T cells in synovial fluid have a significantly greater drug binding rate compared to those in blood [45].

Moreover, the researchers sought to ascertain whether the T cells present in the bloodstream that bind to drugs were analogous to the expanded cells observed in the joints of ICI arthritis. Researchers categorized CD8+ T lymphocytes into three categories based on blood and synovial fluid samples, separating T cells into groups that were either bound to drugs or not, and carried out extensive TCR sequencing [22]. The analysis results indicate significant similarity between the TCR sequences of drug binding cells present in synovial fluid or blood. This suggests that T cells present in synovial fluid originate from the proliferation of T cells that interact with ICI drugs in the bloodstream (Fig. 2A).

To sum up, the characteristics of T cells in patients with ICI arthritis are shown in Table 2. The findings indicate that a notable amount of T cells in individuals with ICI arthritis show attachment to ICI medications. The interruption of gene activity in T cells during ICI arthritis is probably caused by ICI medications. In light of the experimental findings, we put forth the following line of reasoning: following the release of ICI drugs into the bloodstream, they bind to T cells in the bloodstream, thereby altering their gene expression and exhibiting characteristics that are strikingly similar to those observed in ICI arthritis T cells. Following this, the T cells, which have lost function due to the mentioned processes, migrate into the synovial fluid, thus starting the progression of ICI arthritis.

**Table 2:** The characteristics of T cells in patients with ICI arthritis.

Characteristics of T cells		ICI arthritis	Autoimmune arthritis
**Number of T cells**	Regulatory cells T peripheral helper cell Mature T cell	Lower Lower Higher	Higher Higher Lower
**Gene expression rate**	Cytotoxicity Dysfunction Proliferation Chemokines Chemokine receptors	Higher Higher Higher Higher Higher	Lower Lower Lower Lower Lower
**Anaerobic**		Higher	Lower
**IFN I sensitivity**		Higher	Lower
**Bind to ICI drugs**		Yes	No
**TRBV gene**		Heterozygous	Homozygous

## 5. Mechanisms of T cells in the development of ICI arthritis

### 5.1. The disorder of T cell composition and gene expression

Individuals suffering from ICI arthritis exhibit an imbalanced T cell makeup, featuring a significant increase in harmful CD8+ T cells and a substantial decrease in Tregs with immunosuppressive properties. This alteration is exclusive to patients with ICI arthritis [35]. This cellular level change inevitably leads to overactivation of the patient’s immune system, which explains why the immune system function of ICI arthritis patients is dysregulated at the cellular level.

Distinct phenotypic features are also observed in T cells of patients with ICI-induced arthritis. The gene expression profiles of T cells in ICI arthritis patients differ from those observed in RA/PsA patients. In particular, patients with ICI arthritis show increased levels of CD38 and HLA-DR, along with decreased levels of CD127 [45]. Disruption in gene expression can negatively affect T cells, leading to various harmful consequences. To sum up, the disruption of gene activity can lead to the formation and malfunction of T cells, along with an incapacity to operate effectively. Moreover, it may result in heightened T cell resilience against ICI therapy. This offers a detailed explanation at the molecular level for the immune system dysfunction seen in individuals with ICI arthritis.

**Figure 3.**
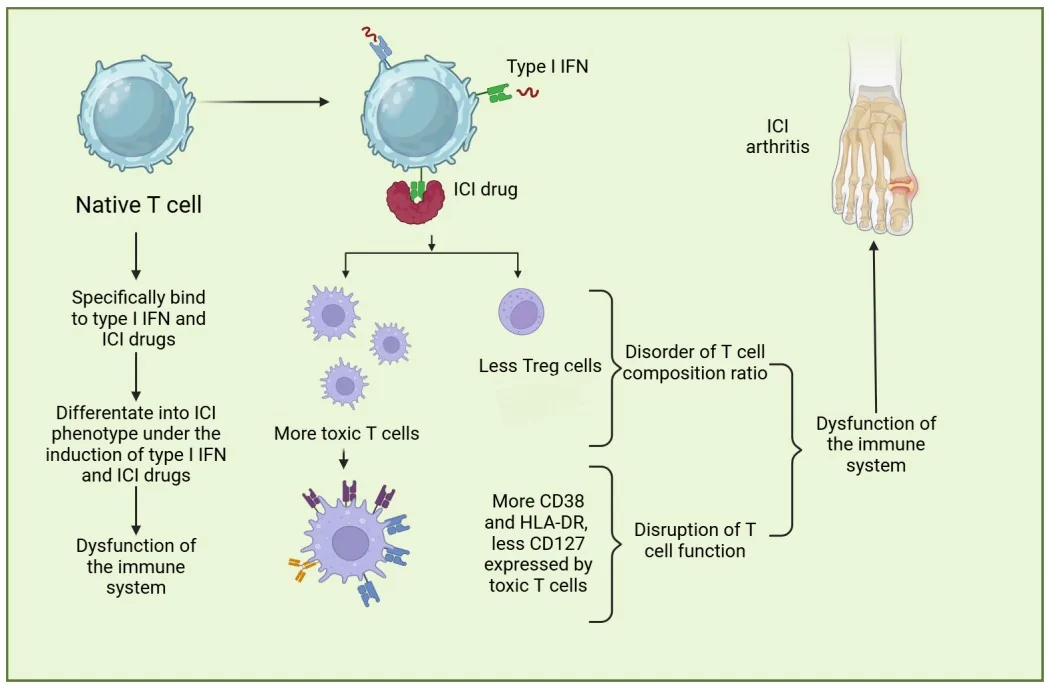
**Mechanisms of T cells in the development of ICI arthritis**. T cells differentiate and mature under the induction of type I IFN and ICI drugs, resulting in variations in gene expression and composition. The disorder of T cell composition and gene expression is the fundamental cause of ICI arthritis.

The aforementioned two aspects of T cell composition and function, in conjunction with other factors, provide a comprehensive explanation for the onset of ICI arthritis in patients. Concurrently, these represent the most substantial alterations in the bodies of patients with ICI arthritis, exerting a pivotal influence on the pathogenesis of this condition (Fig. 3).

### 5.2. Type I IFN and ICI drugs induce the differentiation of T cells into the ICI phenotype

At present, T cells in ICI arthritis undergo changes in their composition and gene expression. Further investigation is required to ascertain the source of these alterations. T cells may differentiate and mature due to the influence of type I IFN and ICI medications, leading to alterations in gene expression and composition, which could be a plausible explanation.

On the one hand, the strong induction effect of type I IFN on T cells has been confirmed in previous experiments. Type I IFN has the ability to prompt immature T cells to develop into T cells exhibiting unique ICI traits. Furthermore, T cells in patients receiving ICI have demonstrated heightened sensitivity to type I IFN, likely as a result of enhanced positive feedback regulation. This is evidenced by the upregulation of type I IFN-responsive proteins in T cells following type I IFN induction, leading to an increased sensitivity to type I IFN. [40]. Under this positive feedback regulation, immature T cells are rapidly induced to become ICI T cells. Notably, this strong induction effect is unique to ICI arthritis: Type I IFN is not normally implicated in the pathogenesis of PsA and is only moderately elevated in a small subset of RA patients [22].

On the other hand, ICI drugs have a potential inducing effect on T cells, and this potential inducing effect is achieved by binding between ICI drugs and T cells. In the experiment, it was observed that T cells bound to ICI drugs exhibited typical ICI T cell phenotypes, consistent with the toxic T cell phenotypes observed in synovial fluid [35, 45]. The aforementioned experimental outcomes lead us to hypothesize that ICI drugs bind to T cells in the bloodstream, thereby modifying their gene expression patterns and exhibiting characteristics analogous to those observed in ICI arthritis T cells. Subsequently, these dysfunctional T cells enter the synovial fluid, thereby precipitating the onset of ICI arthritis. In the future, novel therapeutic strategies may be developed to facilitate the separation of T cell and ICI drugs for joints, with the aim of alleviating the symptoms of ICI arthritis (Fig. 3).

### 5.3. The cytotoxicity of T cells manifests specifically at the joints

It is noteworthy that although T cells are distributed throughout the body, they only exhibit robust pro-inflammatory effects in joint synovial fluid [22, 45]. Meanwhile, the high reactivity of T cells to type I IFN is limited to T cells in synovial fluid. It has been demonstrated that type I IFN has the capacity to induce immature T cells to differentiate into mature T cells. The differentiated T cells display elevated levels of pro-inflammatory activity and exhibit characteristics that are consistent with those observed in T cells associated with ICI arthritis. This process ultimately leads to the development of ICI arthritis. Type I IFN is typically not implicated in the pathogenesis of PsA and is only moderately elevated in a small subset of RA patients. It is possible that there is a strong relationship between the heightened pro-inflammatory impact of T cells in joints and their vulnerability to type I IFN in this area. This observation indicates that amplification and type I IFN are key factors in triggering T cell cytotoxicity, which is responsible for the capacity of T cells to cause tissue damage in ICI arthritis. It can be posited that the inhibition of the type I IFN response represents a potential avenue for the treatment of ICI arthritis. Nevertheless, this approach carries the potential risk of compromising the anti-tumor response to ICI treatment.

### 5.4. Genetic mutations in T cells increase the risk of ICI arthritis

A study demonstrates that the incidence of irAEs following ICI treatment is associated with the genetic predispositions of individual patients. Different genetic mutations result in the formation of T cell membrane receptors that are specific to individual patients. As T cells mature in the thymus, they generate autoantigens using specific surface receptors through VDJ recombination, a process that rearranges V, D, and J gene fragments of immunoglobulins [46]. It is possible that not all of the autoantigens will be eliminated during the process of thymic negative selection. Following existing models of T cell development, T cells with autoreactive TCRs are either removed through negative selection in the thymus or stay inactive in the peripheral area. One theory suggests that ICI therapy may revive these T cells, leading to a notable rise in T cell counts after receiving ICI treatment. This also elucidates the mechanism by which ICI-treated patients develop arthritis, as ICI therapy impairs the efficacy of thymic negative selection, resulting in an imbalance in T cell generation [40].

From a clinical symptom perspective, ICI arthritis is very similar to autoimmune arthritis. Regarding T cell function, the involvement of T cells in initiating autoimmune inflammation and ICI joint disease is similar as well. Mobilized T cells proliferate extensively in the blood, enter the joints from local blood vessels, secrete a large amount of pro-apoptotic factors, and induce apoptosis of joint tissue cells. T cells also produce lactic acid through aerobic respiration, which has a toxic effect on tissue cells. However, treating ICI arthritis by inhibiting the activity of T cells is not acceptable, as fully functional T cells are crucial in combating tumor immunity.

## 6. Potential treatment strategies for ICI arthritis

### 6.1. Targeted selection of ICI therapy: monotherapy or combination therapy

**Figure 4.**
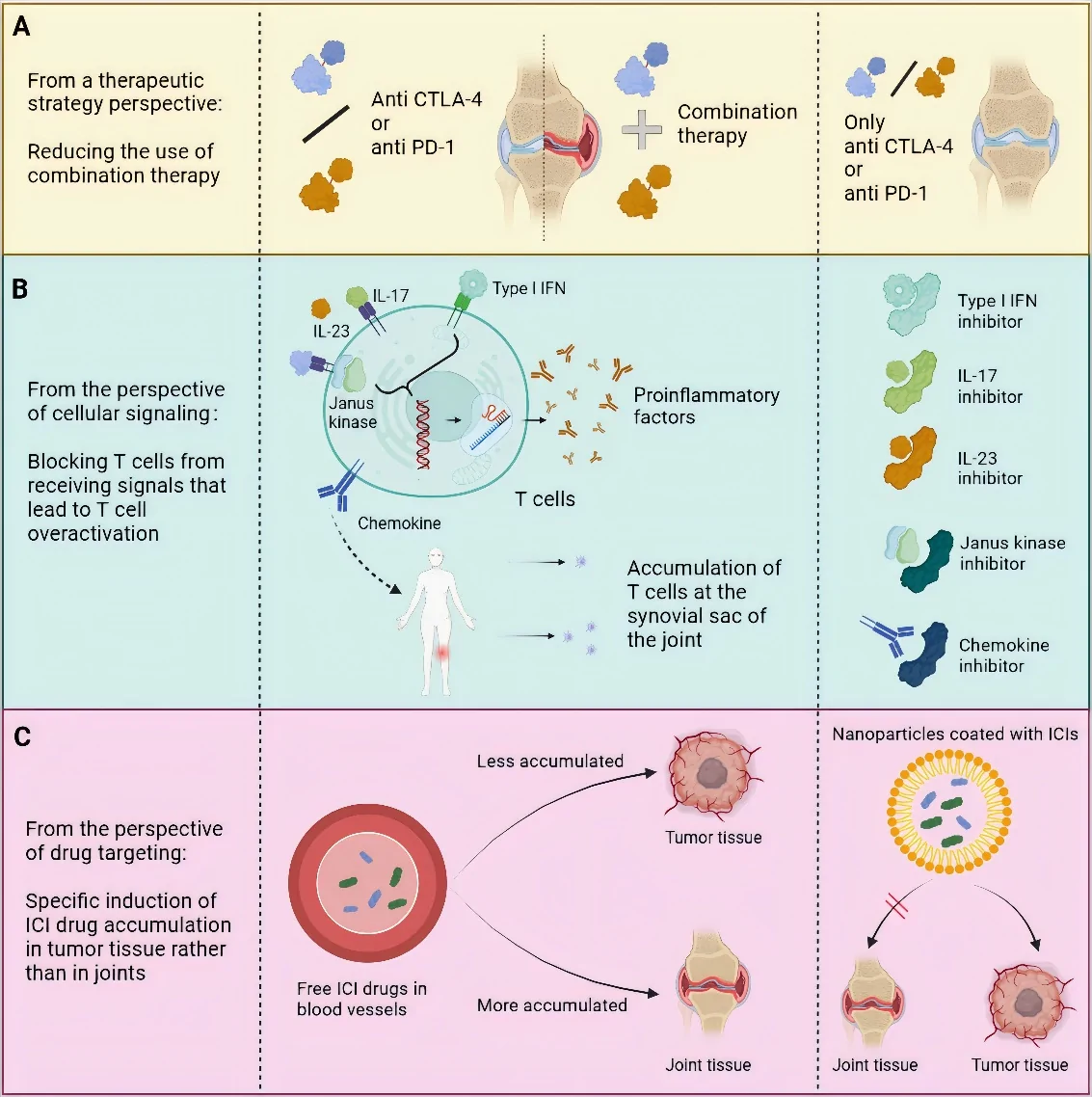
**Potential treatment strategies for ICI arthritis**. (A) Reducing the use of combination therapy; (B) Blocking T cells from receiving signals that lead to T cell overactivation; (C) Specific induction of ICI T cells’ accumulation in tumor tissue rather than in joints.

Studies have shown that the joint action of PD-1 and CTLA-4 can boost the immune system’s reaction and extend duration [47-50]. For example, the combination of anti-PD1 and anti-CTLA4 ICI has been demonstrated to markedly enhance the objective remission rate of melanoma (from 43.7% to 57.6%) [51, 52]. However, there is substantial proof indicating that patients who receive combined ICI therapy have a higher probability of experiencing ICI-induced irAEs, such as ICI arthritis [51, 53] (Fig. 4A). Choosing a single drug therapy to reduce the severity of irAEs is acceptable in cases of low tumor malignancy, but in cases of high tumor malignancy, choosing a single drug therapy may not achieve the required immune strength. Therefore, the choice between monotherapy and combination therapy must be carefully considered.

Presently, immunotherapy is not a widely utilized modality, largely due to the significant challenge posed by the necessity for clinical modelling [47]. While preliminary preclinical studies of immunotherapy have yielded promising results in simulated clinical settings, numerous other noteworthy preclinical findings remain clinically unverified due to the intricate and particular nature of human biological processes. Cutting-edge research is currently underway to improve the controllability and detectability of combination therapy to monitor treatment response and prevent irAEs. With regard to the predictability of combination therapy, Lyu et al. employed machine learning algorithms to develop a model that serves as a predictive tool for combination therapy response, accurately delineating the interrelationship between immune response, thyroid hormone, and cholesterol metabolism. In addition, PA, LDH and APOC3 were proposed as potential response biomarkers for combination therapy [51]. The results of these studies may serve as reliable models for the clinical application of combination therapy, thereby reducing the incidence of irAEs.

### 6.2. Targeted anti-interleukin therapy for treating ICI arthritis while maintaining immune system activity

As mentioned earlier, the function of T cells in ICI arthritis is similar to what is seen in autoimmune arthritis. However, the treatment approaches for autoimmune arthritis are not suitable for ICI arthritis due to the significant impact of immune activity on ICI therapy [54, 55]. It is therefore imperative that a therapy be developed which is capable of treating ICI arthritis while maintaining immune system activity. Anti-interleukin (IL) therapy is a treatment that can simultaneously satisfy two conditions.

At present, the most commonly utilized pharmacological agents are those that inhibit IL-17A and IL-6R. Secukinumab, a drug that blocks IL-17A, has been shown to reduce the occurrence of autoimmune and inflammatory conditions caused by IL-17A [56]. Secukinumab effectively decreases inflammation by targeting IL-17A and inhibiting its activity. Studies have shown that the involvement of TNF-α and IL-17 plays a significant role in the development of inflammatory arthritis, skin conditions, and various autoimmune diseases [56, 57]. Cytokine-mediated pathways are activated by the interaction of IL-23 and IL-17 with different tissue-specific cells, leading to the emergence of intense inflammatory reactions, bone erosion, and the creation of abnormal synovial endometrial complexes. In a treatment targeting two patients with ICI arthritis, the researchers observed that the use of conventional drugs (such as steroids) was not efficacious. Subsequently, the researchers employed anti-IL-17A inhibitors, which resulted in a notable reduction in arthritis symptoms. Furthermore, the tumor ceased to grow following treatment with anti-IL-17A inhibitors, thereby demonstrating that anti-IL-17A inhibitors can effectively treat ICI arthritis while maintaining immune system activity [58] (Fig. 4B).

Another type of anti-IL drugs is IL-6R inhibitors. IL-6R inhibitors block the binding of IL-6 to cells by specifically binding to IL-6 receptors, thereby inhibiting the action of IL-6. IL-6 is also a key cytokine that mediates inflammatory responses [59]. The differentiation of immature CD4 T cells into mature T cell depends on IL-6 [60-63]. IL-6 plays a significant role in the development of various autoimmune conditions such as RA, giant cell arteritis, systemic sclerosis-related interstitial lung disease, and cytokine release syndrome. It is possible that not all of these autoantigens will be eliminated during the process of thymic negative selection. Current models of T cell development suggest that T cells expressing autoreactive TCRs are either deleted during negative selection in the thymus or become quiescent in the periphery. One theory suggests that ICI therapy may revive these T cells, resulting in a significant rise in T cell count following ICI treatment. This also explains why ICI arthritis patients produce excessive amounts of CD8+ T cells, as ICI treatment reduces the effectiveness of thymic negative selection, leading to imbalanced T cell generation. Clinical trials have shown that utilizing anti-IL-6R antibodies can lessen the intensity of inflammatory reactions in various illnesses such as inflammatory arthritis, hepatitis/cholangitis, PMR, nephritis, systemic sclerosis, and central nervous system vasculitis. Of these, anti-IL-6R has shown the greatest efficacy in treating ICI arthritis [64]. It can be reasonably proposed that targeting IL-6R may prove an efficacious method for treating a number of different irAEs without impeding anti-tumor immunity. At present, further clinical trials are underway to assess the efficacy of this drug in reducing the inflammatory response by blocking IL-15 receptors (Fig. 4B).

The current research on ILs is restricted to specific signaling pathways and does not encompass the full immunological landscape of irAEs, which represents a limitation [64]. It would therefore be prudent for future research on ILs to commence with an in-depth examination of the complete immunological characteristics of irAEs, in order to mitigate potential risks. At present, there is still a lot of research to be done targeting interleukins. In addition to IL-17 and IL-6, pro-inflammatory interleukins such as IL-1, IL-2, IL-12, IL-18, etc. have different structures and play different roles in regulating immune system function [65, 66]. Whether there are other interleukin inhibitors that have similar effects to IL-17 and IL-6 remains to be investigated.

### 6.3. Targeted signaling pathway therapy: JAK inhibitors maintain T cell activity and anti-tumor ability

In addition to anti-IL therapy, intervention in the signaling pathway of T cell maturation may also prove an effective strategy for achieving the simultaneous maintenance of immune activity and reduction of inflammatory response. At present, the two most commonly intervenable signaling pathways are the TNF signaling pathway and the JAK signaling pathway. Inhibition of either of these pathways has been demonstrated to result in a reduction in the inflammatory response. Nevertheless, the inhibition of the TNF signaling pathway may result in a reduction in immune system activity, accompanied by a simultaneous reduction in the inflammatory response. Conversely, the inhibition of the JAK signaling pathway does not result in a substantial reduction in immune activity [67, 68].

The current debate surrounds the utilization of TNF inhibitors in irAE [69-71]. Nevertheless, the use of TNF inhibitors in irAE is becoming increasingly controversial. A study has demonstrated that CD8+ T cells in ICI-treated patients with arthritis exhibit downregulation of the TNF signaling pathway, resulting in reduced TNF release compared to those receiving ICI treatment without disease [72, 73]. Continued use of TNF inhibitors in conditions of reduced TNF undoubtedly exacerbates the immune dysfunction caused by insufficient TNF. Meanwhile, clinical trials have also shown that patients treated with TNF inhibitors have reduced neutrophils and are more susceptible to infection [74, 75]. Therefore, the widespread use of TNF inhibitors can alleviate symptoms, but also greatly reduce the activity of the immune system, which cannot achieve the anti-tumor goal.

Identifying different treatment approaches that can prevent musculoskeletal inflammation caused by ICI, while still maintaining anti-cancer effects, is crucial to address the current clinical requirements for arthritis management. The potential for tofacitinib, a JAK inhibitor, to become a therapeutic medication is clear [76, 77]. Tofacitinib has previously demonstrated successful usage in a patient with arthritis irAE who received ICI treatment, as well as in a group of GC refractory myocarditis irAE cases [35]. In laboratory tests, it was shown that tofacitinib did not decrease the production of immune-activating substances from T cells in individuals with ICI arthritis. Moreover, the existence of this medication did not hinder the ability of T cells to fight tumors in individuals with ICI arthritis. Tofacitinib has the potential to play a role in reducing inflammation while maintaining the anti-tumor capabilities of T cells. Therefore, it can be inferred that tofacitinib shows promise as a potential new medication for treating anti-ICI arthritis. Further research is required to confirm this hypothesis [35, 78] (Fig. 4B).

In addition to tofacitinib, the potential of other novel JAK inhibitors is also being investigated. For example, the book 2-amino-4-phenylaminopyrimidine JAK/HDAC inhibitors can block both the JAK and HDAC pathways at the same time, making them more effective in treating cancer [79, 80]. The HDAC enzyme is often a focus in the creation of medications to combat tumors’ inhibitors are unable to activate the JAK/STAT classical signaling pathway because of the presence of the leukemia inhibitory factor receptor (LIFR)-JAK-STAT feedback loop, which results in the upregulation of LIFR feedback. This severely limits their therapeutic efficacy in solid tumors. Qian et al. Researchers have discovered a new inhibitor that targets both JAK/HDAC, effectively blocking the JAK-STAT and HDAC pathways at the same time. Both laboratory and animal studies have shown that this blocker has beneficial treatment outcomes for both solid tumors and blood cancers. Furthermore, it has been shown to effectively compensate for the poor therapeutic effect of single target HDAC inhibitors in solid tumors. It has the capacity to inhibit HDAC3/6 and JAK at the nanomolar level, while demonstrating highly selective inhibitory activity against JAK. Consequently, the compound is capable of simultaneously blocking the JAK-STAT and HDAC signaling pathways, thereby inducing tumor cell apoptosis and exhibiting good anti-tumor proliferation activity on solid tumor cells (A549) and hematological tumor cells (HEL) [79]. Most notably, in diverse xenograft tumor models, the drug has demonstrated superior or equivalent anti-tumor efficacy relative to conventional JAK inhibitors. The results indicate that the dual-target inhibitor exerts synergistic anti-tumor effects and a more expansive anti-tumor spectrum. Thus, the groundwork has been laid for the development of JAK-HDAC inhibitors, with important implications for targeted cancer treatments.

### 6.4. Targeted T cell differentiation therapy: specific inhibiting IFN sensitivity of T cells in synovial fluid

The induction of IFN exerts a more pronounced effect on T cells present in the synovial fluid of the joint. Furthermore, IFN induction has been observed to differentiate naive T cells into more mature and more effective T cells. The combination of these two points allows for the formulation of a hypothesis that T cells present in the synovial fluid of the joint are susceptible to stimulation by IFN, resulting in the development of mature T cells with pronounced pro-inflammatory properties, which ultimately lead to the onset of ICI arthritis. Exploring the cause of T cells’ heightened sensitivity to TNF in the synovial fluid of the joint could prove advantageous. This could include exploring its potential connection to the stimulation of the synovial microenvironment. The inhibition of T cell sensitivity to TNF in the synovial fluid of the joint can effectively reduce joint inflammation while maintaining normal T cell function in other regions (Fig 4B).

The first in class type I IFN inhibitor, Saphnelo has been approved for marketing in recent years. Consequently, current research should concentrate on enhancing the efficacy and delivery methods of drugs. The dense cartilage and lack of vascular structure characteristic of arthritis present significant challenges to drug delivery to the affected area [81-83]. Developing more precise delivery systems can lead to successful drug delivery [83]. The composition of the arthritis cartilage microenvironment consists primarily of compact type II collagen and proteoglycans, forming a barrier that hinders targeted treatment of chondrocytes. Studies indicate that targeting particular molecules to address cartilage elements could offer fresh approaches for managing arthritis [84, 85]. Studies indicate that WYRGRL, a peptide that targets collagen II, significantly boosted the in vivo cartilage targeting effectiveness of nanoplatforms functionalized with peptides by around 72 times [86]. It can therefore be concluded that by specifically binding WYRGRL to type II collagen, drug-loaded particles are able to aggregate in articular cartilage tissue, thereby achieving targeted drug delivery.

### 6.5. Targeted T cell migration therapy: increasing the accumulation of T cells in tumors rather than joints

As mentioned earlier, individuals without arthritis also show some level of T cell cytotoxicity and anaerobic glycolysis. This occurrence is particularly noticeable in individuals with ICI arthritis. The synovial bursa of ICI arthritis patients contains a significantly higher number of biologically active T cells than is typical, which results in an excessive accumulation of pro-inflammatory factors and glycolytic waste in the joint synovial capsule of ICI-irAE patients [87, 88]. It may therefore be possible to prevent the accumulation of activated T cells in the joints [87, 89].

Intricate proteins such as dendritic cell surface chemokines, selectins, integrins, and other chemical mediators facilitate the movement of T cells within the body at a microscopic scale. The specific chemokine receptors expressed by T cells can assist T cells in recognizing these mediators, thereby enabling T cell migration to specific regions of the body [90, 91]. This provides an explanation at the molecular level as to why T cells accumulate in the synovial capsule. The creation of chemokine receptor blockers on T cells’ surface could be an efficient way to obstruct the identification of T cells and controlling proteins, ultimately slowing down the specific migration of T cells and decreasing their presence in joints [88] (Fig. 4C).

### 6.6. Targeting other immune checkpoints: Lag-3, Tim-3, and TIGIT have lower immune response

At present, popular immune checkpoints consist of CTLA-4 and PD-1. It is undeniable that therapies targeting them have strong anti-tumor effects, but they can also cause abnormal excitation of the immune system. New immune checkpoints have been reported, including lymphocyte activation gene 3 protein (Lag-3), hepatitis A virus cell receptor 2 (HAVcr-2; also known as TIM3) and T cell immune receptor with immunoglobulin and ITIM domains (TIGIT) [92]. What sets them apart is their mildness in comparison to the traditional CTLA-4 and PD-1, without causing an overactive immune reaction [92].

Lag-3 is a molecule that becomes more active in activated CD4 and CD8 T cells as well as subsets of natural killer (NK) cells [92]. Recent experiments have shown that Lag-3 can inhibit effector T cells and promote the function of Treg cells. Therefore, anti-Lag-3 therapy can activate the immune system and achieve anti-tumor function [93, 94]. Nevertheless, the precise manner in which Lag-3 transmits signals in these various T-cell groups to produce its suppressive impact remains uncertain. There are precedents for the clinical use of Lag-3 inhibitors for anti-tumor treatment, and there have been no treatment-related irAEs in relevant clinical trials [92, 94, 95]. Despite demonstrating some therapeutic benefits in clinical settings, the way in which Lag-3-Ig controls the anti-tumor reaction remains unknown. The research project on Lag-3 is still in progress.

T cell immunoglobulin 3 (Tim-3) is a cell surface molecule selectively expressed on CD4 helper and CD8 cytotoxic T cells that produce IFN-γ [96]. Tim-3 plays a crucial role in controlling the activity of effector T cells, with recent findings indicating that the Tim-3 tail has the ability to engage with various elements of TCR complexes. [97]. Participating in various signal recognition pathways on effector T cell membranes, it can potentially suppress dendritic cell activation by serving as a molecular companion to HMGB1, a crucial protein in numerous pathological and physiological functions [98]. Recent research has demonstrated that blocking the Tim-3 pathway can effectively boost the immune system and be a promising target for treating conditions like arthritis [98, 99].

TIGIT, a member of the Ig superfamily, is found exclusively on immune cells and functions as a co-inhibitory receptor on these cells [100-102]. TIGIT directly affects T cells and NK cells through its interaction with CD155 and CD112 and can also inhibit immune responses indirectly by activating CD155 on DCs [103-105]. During clinical trials involving patients with melanoma, combining TIGIT and PD-1 led to enhanced tumor growth inhibition and decreased cytokine release [106, 107]. Likewise, the combined inhibition of TIGIT and PD-L1 demonstrated a cooperative impact in the CT26 tumor model in mice. TIGIT not only collaborates with PD-1, but also with Tim-3, hindering the beneficial anti-cancer reaction. Hence, combining TIGIT inhibition with PD-1 or TIGIT with Tim-3 could enhance the body’s ability to fight against tumors and lead to tumor shrinkage. Together, this information indicates that TIGIT collaborates with additional co-inhibitory molecules to inhibit effector T cell reactions and encourage T cell impairment.

Anderson et al. suggested a different approach where CTLA-4 and PD-1 are seen as the initial immune checkpoints, mainly tasked with preserving self-tolerance and controlling T-cell growth in lymphoid organs, while Lag-3, Tim-3, and TIGIT are viewed as the subsequent immune checkpoints with distinct functions in managing immune reactions [92]. Compared to the first-tier immune checkpoints, Lag-3, Tim-3 and TIGIT have a narrower range of action, weaker effects and are safe. They therefore have the potential to serve as a new generation of targets for immune checkpoint inhibitor therapy. At the same time, however, they also have some problems because they do not activate the immune system as much as the first-tier immune checkpoint, which compromises their efficacy in tumor elimination [108]. Perhaps a combination with traditional therapies such as chemotherapy and radiotherapy can compensate for this drawback.

### 6.7. Tumor targeted ICI therapy: Probody drug delivery system specifically recognizing the tumor microenvironment

A potential approach to minimize the negative impacts of irAE involves specifically focusing on ICI antibodies and restricting their overall presence in the body. An exemplar of a targeted checkpoint inhibitor antibody is CytoMX’s Probodies [109]. By identifying the tumor microenvironment, it successfully triggers the immune system as intended [110-112].

The Probody method of delivering drugs is made up of three separate parts: ICIs, peptides that hide the ICIs’ light chain at the beginning, and peptides that can be broken down by proteases [110, 111]. There are many specific molecules in the tumor microenvironment, such as caspases, that are unique to the tumor microenvironment. In healthy tissues, the Probody system remains largely intact, blocking target binding and maintaining the expected long circulating half-life of monoclonal antibody therapy. When Probody is introduced into the tumor microenvironment, tumor-associated proteases cleave substrate linkers, releasing masking peptides that allow antibodies to bind to target antigens, thereby specifically activating the immune system in tumor tissue [109-111]. Although Probody has not yet entered clinical development, pre-clinical results appear promising. Studies conducted in a lab setting have demonstrated that Probody without a mask exhibits strong, dose-dependent ability to kill tumors, whereas the presence of masking molecules can decrease cytotoxicity by over 100,000 times. The Probody, when concealed, displayed notable effectiveness against tumors in the HT29 transplantation model in mice that were infused with human PBMCs, achieving complete regression of tumors at a dosage of 1.5 mg/kg after showing significant activity at 0.5 mg/kg [109]. The findings from this research indicate that Probody shows promising safety and effectiveness against tumors.

The Probody drug delivery system is currently still in the experimental phase. In order to achieve effective targeted therapy, it is first necessary to identify specific proteases present in the tumor microenvironment [113, 114]. Due to the unique characteristics of tumor tissue, the marker proteases in its microenvironment can be highly diverse and undergo changes over time [115-117]. Consequently, one of the challenges that the Probody drug delivery system must address is the effective targeting of tumor tissue over an extended period of time.

## 7. Conclusion and prospect

This review examines the role of T lymphocytes in the progression of ICI arthritis. Arthritis is a major complication linked to ICI treatment, posing a barrier to the widespread use of ICI therapies. Our main goal in the analysis was to investigate the unique features of T cells in patients with ICI arthritis. These encompassed factors related to T cell activation, quantities and developmental stages of T cells, secretions and immune responses of T cells, the specific binding capacity of ICI arthritis T cells to anti-PD-1 medications, and the role of TCR in inducing irAEs. Proposed targeted solutions are based on these characteristics.

Various drugs, such as NSAIDs, GC, SSZ, MTX, HCQ, and TNF inhibitors, are currently used to treat ICI arthritis. Currently, there are emerging pharmaceuticals, including JAK inhibitors, anti-IL-6R agents, and anti-IL-17A antibodies, that show promise in the treatment of ICI-arthritis. A number of these medications are traditional treatments for RA, and they are ineffective in the treatment of ICI arthritis. This may be due to their inability to effectively enhance the inflammatory reaction (like traditional anti-RA medications) or their ability to suppress the immune system’s function (like TNFi). Many research studies have indicated that JAKi, anti-IL-6R, and anti-IL-17A have the potential to be highly efficacious therapeutic agents. Furthermore, ongoing investigations have proposed that these three types of medication have minimal impact on immune system function. It is logical to conclude that these agents could potentially be a novel class of medications for managing ICI arthritis.

Although there is a significant amount of evidence pointing to the crucial involvement of T cells in the development of ICI arthritis, our knowledge of the entire pathogenesis of ICI arthritis is still lacking. Exploring the origins of T cell-induced ICI arthritis at the cellular and molecular levels is crucial, as well as suggesting potential research approaches. The review provides an overview of the characteristics of T cells in patients with ICI arthritis. These include an improved capability of binding to ICI drugs, enhanced production of chemokines and chemokine receptors, special T cell receptors on irAE-inducible T cells, higher IFN sensitivity of T cells, elevated count of T lymphocytes in the bloodstream, and a dysregulated proportion of T peripheral helper cells. In light of these findings, we proceeded to investigate potential treatment strategies for ICI arthritis (Table 3). Strategies to address this issue involve minimizing the use of combined ICI treatments, utilizing Anti-IL to manage ICI arthritis without compromising immune function, employing JAK inhibitors to preserve T cell activity and anti-cancer capabilities, targeting IFN sensitivity in T cells within synovial fluid, enhancing T cell accumulation in tumors instead of joints, introducing less potent immune checkpoint inhibitors, and focusing on selectively targeting ICI antibodies to reduce systemic impact.

The findings from this research will guide the planning of upcoming clinical trials, ultimately aiding in the creation of novel medications that focus on these specific processes. One suggestion is that T cells may be more vulnerable to IFN, which is crucial in the abnormal development of T cells. It is therefore proposed that future research should concentrate on the development of specific inhibitors of type I IFN and targeted delivery strategies for the delivery of this inducer to joints. The mechanism of T cells in ICI arthritis also has a significant impact on clinical practice. Our research has elucidated the immune system disorders in ICI arthritis patients at both the cellular and molecular levels. It is therefore imperative to take measures to prevent other immune reactions, such as allergic reactions or other autoimmune diseases, during the course of treatment. Moreover, the influence and particular mechanisms of genetic polymorphism on ICI arthritis have been elucidated. It is therefore imperative that, when applying ICI therapy to treat patients, individual differences are observed, and personalized treatment plans are adopted.

**Table 3:** Potential treatment strategies for ICI arthritis.

Treatment perspectives	Treatment strategies	Drugs
**Improvement of overall therapy**	Reducing the use of anti CTLA-4 and anti PD-1 combination therapy	/
**Reduce inflammatory factors**	Specific inhibition of interleukin	IL-17A inhibitor or IL-6R inhibitor
	Specific inhibition of JAK signaling pathway	JAK signaling pathway inhibitor
		The novel 2-amino-4-phenylaminopyrimidine JAK/HDAC dual target inhibitors
**Reduce abnormal differentiation of T cells**	Specific inhibition of the induction effect of type I IFN on T cells	Type I IFN antagonist or type I IFN receptor inhibitor
**Reduce the aggregation of T cells at joints**	Reduction of T cell migration mediated by inflammatory factors	Chemokine receptor blockers
**New immune checkpoint**	Using immune checkpoints with lower immune response	Lag-3, Tim-3, TIGIT
**Tumor targeted ICI therapy**	Probody drug delivery system specifically recognizing the tumor microenvironment	Probody

Notwithstanding the development of certain drugs and treatment plans for ICI arthritis, our attention to the treatment of ICI arthritis remains inadequate, and there is a paucity of interest in new trials related to ICI arthritis. The dearth of available treatment options underscores the necessity of developing effective methods for predicting and preventing ICI arthritis. This is an emerging field, and the prevention of ICI arthritis can be approached from the following perspectives: (a) The detection of the T cell composition ratio in ICI-treated patients is a key objective. As previously stated, an imbalance in T cell composition can disrupt the body’s immune balance, potentially leading to autoimmune reactions, including ICI arthritis. (b) The detection of T cell expression proteins in ICI-treated patients may provide insight into gene expression disorders in T cells, which could result in significant differences in the quantity and proportion of expressed proteins compared to normal T cells. (c) The detection of T As previously stated, some patients exhibit greater disorder in CDR regions of T cell surface receptors, rendering them more susceptible to developing ICI arthritis following ICI treatment. (d) The sensitivity of T cells to IFN can be determined by hybridizing the patient’s T cells with fluorescent protein-labelled IFN, and increased sensitivity may be indicative of irAEs.

Exploring the precise role of T cells in ICI arthritis and drug advancement through future research would be advantageous. Several questions regarding the unique functions of T cells in ICI arthritis remain unanswered: (a) In addition to expressing specific cell surface receptors, what are the differences in metabolism and protein expression between ICI arthritis T cells and autoimmune arthritis T cells. (b) How are T cells activated and become typical ICI T cells after receiving ICI treatment, and which signaling pathways are involved. (c) The specific expression of T cell cytotoxicity at the joint related to certain molecular signals in the joint microenvironment. (d) The migration of T cells is regulated by a series of proteins, so which proteins play a role in the directional aggregation of T cells towards joints. In terms of drug development, the following avenues of research should be pursued: (a) The development of clinical models for different tumors to monitor the response to ICI therapy and prevent immune system overactivation. (b) The research of new drugs that can suppress ICI arthritis while ensuring immune response, such as novel interleukin inhibitors. (c) The development of a new drug delivery system that can deliver drugs through cartilage tissue into the synovial sac.

This review aims to clarify the precise mechanism of T cell-induced ICI arthritis and offer guidance for upcoming studies. The outlook for treatment appears positive as more and more cancer patients are receiving therapy. By elucidating the mechanism of ICI arthritis, it is possible to provide individuals with the knowledge required to mitigate the adverse consequences associated with this condition. By circumventing a multitude of adverse effects, ICI therapy could gain greater acceptance, thereby facilitating advancements in anti-tumor treatments.
